# 

**Published:** 1894-09

**Authors:** 


### CONTENTS—SEPTEMBER, 1894.—VOL. X., NO. 3.

ORIGINAL COMMUNICATIONS—

Traumatic Neuroses: a Reply to Dr. R. M. Swearingen and The Texas Medical Journal. W. B. Outten, M. D. . . . . . . . . . . . . . . . 97
How Shall The Regular Medical Profession Be Organized into Ethical Bodies? Daniel Parker, M. D. . . . . . . . . . . . . . . . . . . . . 108

CORRESPONDENCE—

"Railroad Spine." Letter from John Eric Erichsen . . . . . . . . . 114
Some Surgical Work in San Antonio. R. Menger, M. D., City Phy'n . 115

CURRENT MEDICAL LITERATURE—

Department of Practice of Medicine. So called Spontaneous Combustion. Prof. A. J. Smith, M. D. . . . . . . . . . . . . . . . . . . . . 118
Miscellaneous Abstracts. By Prof. Wm. Keiller, M. D. . . . . . . . 121

SOCIETY NOTES—

The American Public Health Association—22nd Annual Meeting . . . 125
Mississippi Valley Medical Association—20th Annual Meeting . . . . 126

EDITORIAL—

"Railway Spine" . . . . . . . . . . . . . . . . . . . . . . . . . 127
Shall We Abolish The State Medical Association? . . . . . . . . . . 133
Another Texas Medical College . . . . . . . . . . . . . . . . . . . 134
Sic Transit—Demise of the "Southern Medical Review" . . . . . . 135

MEDICAL NEWS AND MISCELLANY—

Prof. Bell, Climate of Texas for Surgical Work, etc. . . . . . . . . . 135

BOOK NOTICES— . . . . . . . . . . . . . . . . . . . . . . . . . . . 137

PUBLISHERS' NOTES . . . . . . . . . . . . . . . . . . . . . . . . 142

